# Applications in Urticaria

**Published:** 1876-08

**Authors:** 


Applications in Urticaria.—Professor Hardy recommends (*Union Med.*, May 20) the following lotion to be applied several times a day in order to allay the itching in urticaria: Chloroform ten, and oil of sweet almonds thirty parts. In obstinate cases he prescribes corrosive sublimate one-tenth to one-seventh of a part, alcohol ten parts, and distilled water ninety parts. He gives also internally alkaline medicines, and if these do not prove efficacious he resorts to arsenic.—*Med. Times and Gaz.*, May 27, 1876.

